# Supplementary material for: Morphological and Molecular Functional Evidence of the Pharyngeal Sac in the Digestive Tract of Silver Pomfret, Pampus argenteus
Source: Int J Mol Sci. 2023 Jan 14;24(2):1663. doi: 10.3390/ijms24021663 (PMC9866116; doi:10.3390/ijms24021663)
Supplement: Supplementary file 1 [file ijms-24-01663-s001.zip › ijms-2058773-supplementary.pdf]

Supplementary Material

1 Supplementary Figures and Tables

1.1 Supplementary Figures

Supplementary Figure S1 De novo transcriptome assembly and annotation of *P. argenteus*.

(A) BUSCO assessment results. (B) Pearson correlation analysis. Ps and E indicate the pharyngeal sac and the esophagus, respectively. (C) The unigenes annotation of seven databases. (D) Venn diagram from annotation results from five databases.

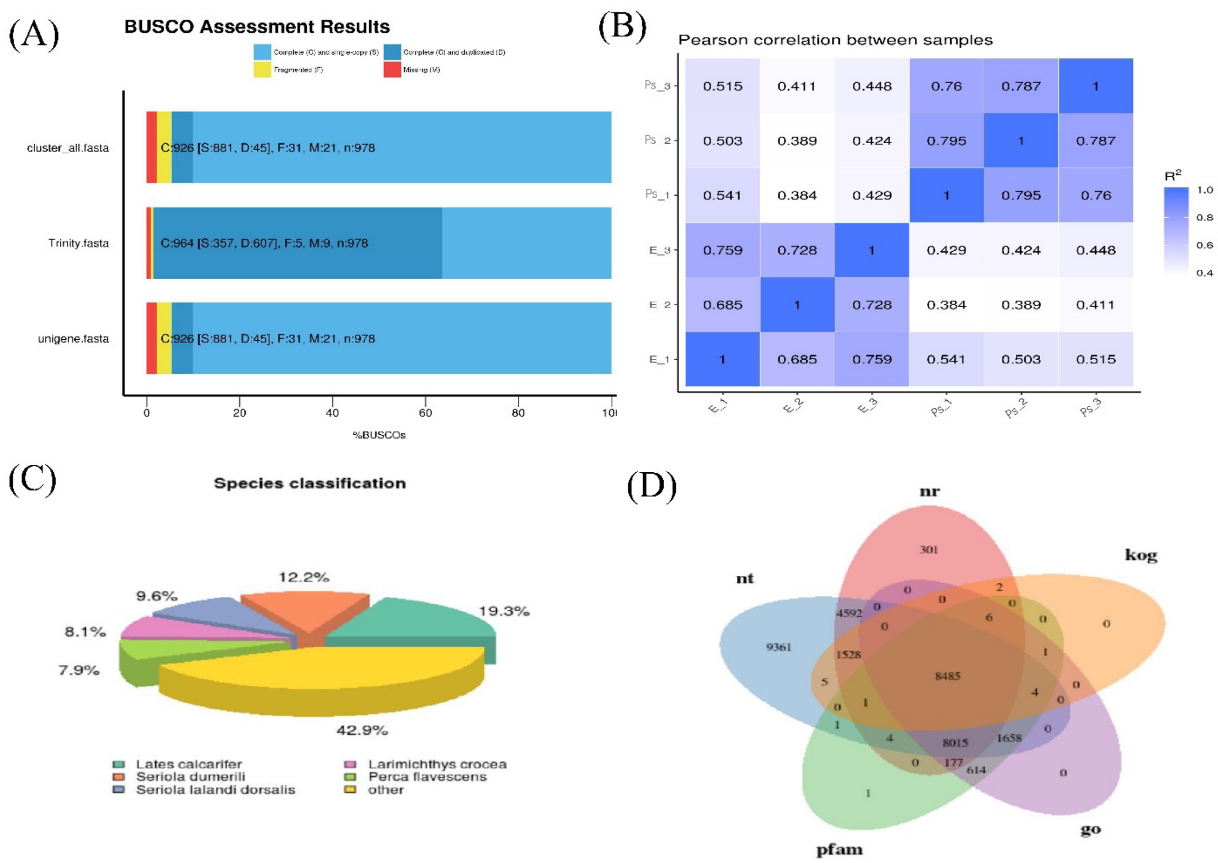

Supplementary Figure S2 Gene Ontology (GO) classifications of all unigenes from pharyngeal sac of *P. argenteus*.

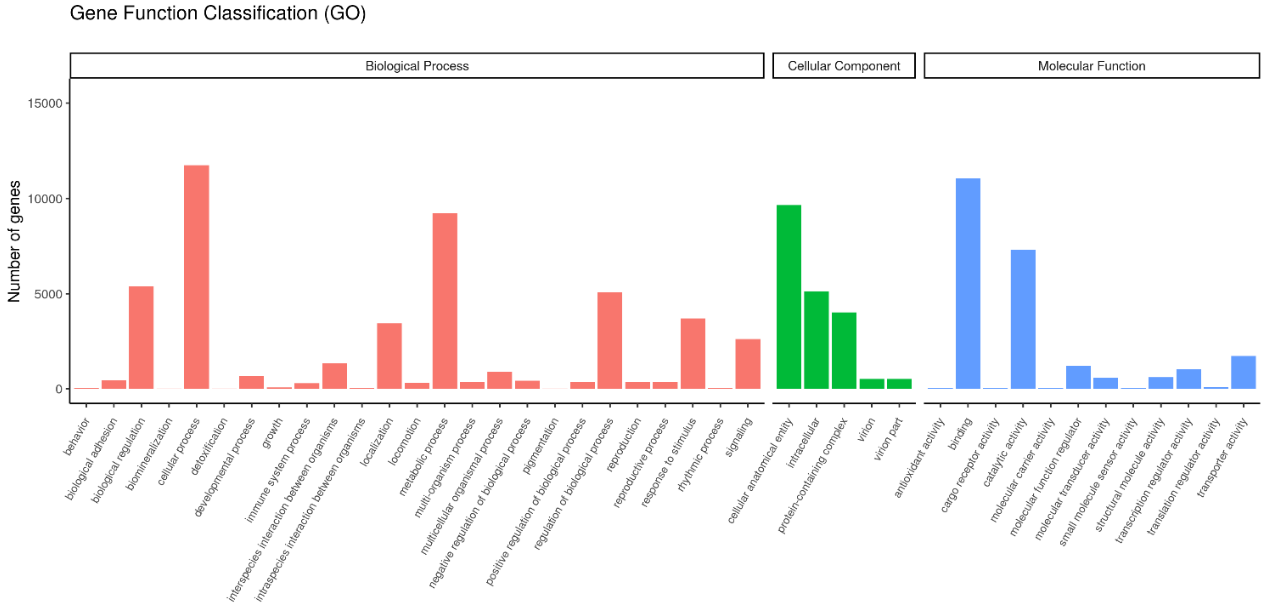

Supplementary Figure S3 Kyoto Encyclopedia of Genes and Genomes (KEGG) classifications.

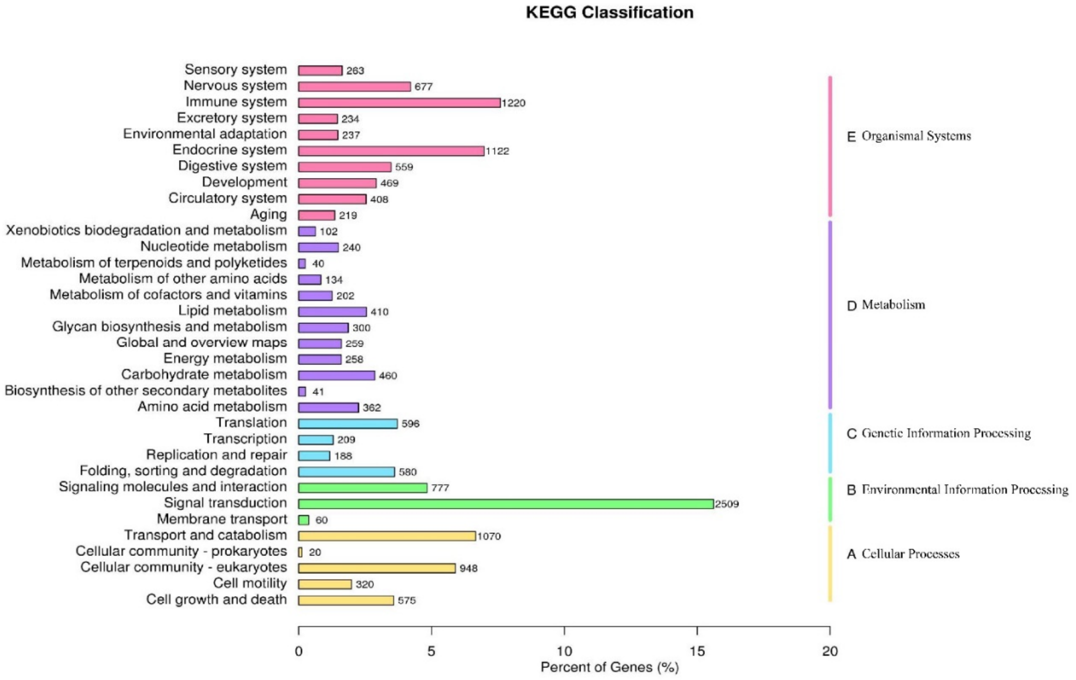

Supplementary Figure S4 Clusters of Orthologous Groups of proteins (KOG) function classification.

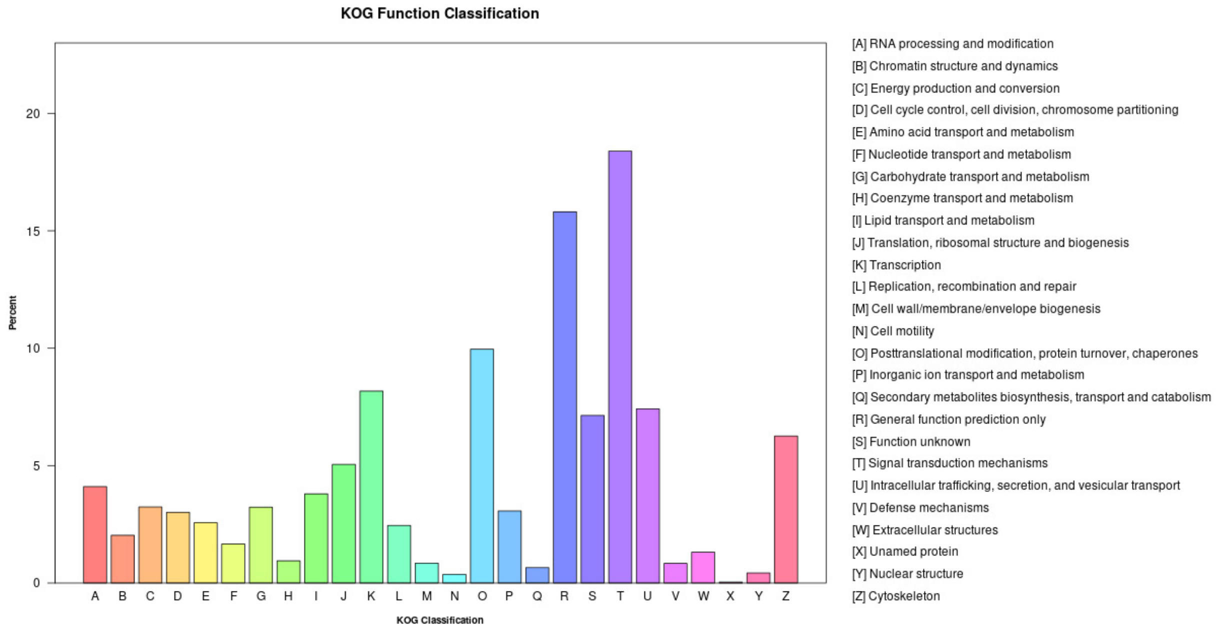

1.2 Supplementary Tables

Supplementary Table S1 Statistics of sample comparison

Sample name	Total reads	Total mapped
E_1	21389250	16150441(75.51%)
E_2	23789510	16915448(71.10%)
E_3	23169591	17136386(73.96%)
Ps_1	19896859	15594954(78.38%)
Ps_2	22190761	17256919(77.77%)
Ps_3	20482075	15453665(75.45%)

Supplementary Table S2 The length distribution of transcripts and unigenes.

The numbers of transcripts and unigenes for different types of length intervals							
Transcript length interval			300-500bp	500-1kbp	1k-2kbp	>2kbp	Total
Number of transcripts			20320	20146	26814	38205	105485
Number of unigenes			12420	9638	8104	11521	41683
**The length distribution of transcripts and unigenes**							
	Min Length	Mean Length	Median Length	Max Length	N50	N90	Total
Transcripts	301	1898	1414	48909	2910	921	200177226
Unigenes	301	1587	896	48909	2788	602	66135292

Supplementary Table S3 Statistics of transcription factors.

Tf Family	KO Name	Description	Log_2Fold Change	Padj	Up/Down
PAX	PAX1/9	paired box protein 1/9	12.753	2.8106E-22	Up
	PAX3/7	paired box protein 3/7	7.0428	0.000019304	Up
ZBTB	KLHL3/1	kelch-like protein 3/1	12.233	3.4936E-18	Up
	KBTBD5/10	kelch repeat and BTB domain-containing protein 5/10	10.973	1.2531E-13	Up
	ABTB2	ankyrin repeat and BTB domain containing 2	3.2785	1.0764E-08	Up
bHLH	MYF4	myogenic factor 4	10	1.0139E-12	Up
	LMYC	L-myc proto-oncogene protein	2.4812	0.00010659	Up
Homeobox	PITX2	paired-like homeodomain transcription factor 2	9.129	5.8639E-18	Up
	HOX4	homeobox 4	5.1656	5.0792E-09	Up

T-box	TBX5	T-box protein 5	-8.2013	4.5989E-44	Down
	TBX4	T-box protein 4	-6.0419	1.4268E-13	Down
ESR-like	ESRRG	estrogen-related receptor gamma	-7.6689	1.6097E-20	Down
CSD	YBX2	Y-box-binding protein 2	-7.5534	6.9171E-41	Down
zf-GATA	GATA5	GATA-binding protein 5	-6.9958	2.7355E-20	Down
	GATA6	GATA-binding protein 6	-6.5897	3.1671E-39	Down

Supplementary Table S4 Part of the DEGs.

Gene Name	Description	$Log_2FoldChange$	Padj	Up/Down
KRT8	keratin 8	4.996	6.47E-06	Up
KRT13	keratin13	9.2607	1.15E-05	Up
MSLN	mesothelin	3.1821	0.001473	Up
MUC5A	mucin 5AC	8.8769	1.73E-23	Up
PGA	pepsin A	-13.229	2.12E-54	Down
GGH	gamma-glutamyl hydrolase	-9.7675	1.9737E-29	Down
AMCase	acidic mammalian chitinase	-12.759	2.18E-67	Down
SLC6A18	solute carrier family 6-member 18	-12.297	7.6475E-20	Down
PRSS2	serine protease 2	-8.707	1.11E-18	Down
PRSS3	trypsin-3-like	-8.9744	1.2839E-06	Down

CTRA	chymotrypsin A	-9.4016	1.4918E-08	Down
CTRB	chymotrypsin B	-9.2122	2.403E-21	Down
CPAA2	carboxypeptidase A2	-8.6516	3.0157E-17	Down
CPAB	carboxypeptidase B	-6.6772	6.6316E-14	Down
CPAA1	carboxypeptidase A1-like	-5.3928	6.9695E-10	Down
AMY	pancreatic alpha-amylase	-9.664	2.3555E-08	Down
APOE	apolipoprotein E	7.132	3.16E-31	Up
APOL3	apolipoprotein L3	6.9701	2.46E-32	Up
LRP1	low-density lipoprotein receptor related protein 1	3.0946	4.62E-16	Up
LDRL	low-density lipoprotein receptor	4.4959	3.67E-07	Up
FASN	fatty Acid Synthase	2.5978	2.69E-09	Up
MRC	mannose receptor C	10.246	4.45E-08	Up
TOLLIP	toll-interacting protein	4.4659	6.4884E-42	Up
TLR3	toll-like receptor 3	3.6502	4.6436E-07	Up
IGHM	immunoglobulin M heavy chain	2.4397	4.8815E-06	Up
MYH	myosin heavy chain	5.7828	2.78E-14	Up
MYLPF	myeloid cell leukemia1	12.158	1.2262E-46	Up
DES	desmin	8.2848	3.7888E-31	Up
MYL4	myosin light chain 4	9.5317	0.000054225	Up

FLNA	filamin A	2.5721	1.19E-07	Up
CLDN7	claudin 7	5.6693	7.17E-13	Up
COL1A	collagen type I alpha 1 chain	2.2579	5.17E-06	Up
COL10A1	collagen type X alpha 1 chain	13.666	3.49E-23	Up
MEP1B	meprin A subunit beta	10.185	7.8035E-44	Up
WNT5	wnt family member 5	2.9135	2.69E-07	Up
WNT2	wnt family member 2	-7.4718	1.58E-22	Down
TBX5	T-box transcription factor 5	-8.2013	4.5989E-44	Down
TBX4	T-box transcription factor 4	-6.0419	1.4268E-13	Down
TBX1	T-box transcription factor 1	3.8038	1.15E-09	Up
PAX9	paired box 9	12.753	2.81E-22	Up
